# Longitudinal changes in cardiac mIBG scintigraphy in mild cognitive impairment with Lewy bodies

**DOI:** 10.1192/bjo.2024.766

**Published:** 2024-12-05

**Authors:** Gemma Greenfinch, Calum A. Hamilton, Paul C. Donaghy, Michael Firbank, Nicola A. Barnett, Louise Allan, George S. Petrides, John-Paul Taylor, John T. O'Brien, Alan J. Thomas

**Affiliations:** Newcastle University Translational and Clinical Research Institute, Newcastle, UK; The Newcastle upon Tyne Hospitals NHS Foundation Trust, Newcastle upon Tyne, UK; and University College London Hospital, London, UK; Newcastle University Translational and Clinical Research Institute, Newcastle, UK; University of Exeter Medical School, Exeter, UK; Nuclear Medicine Department, The Newcastle upon Tyne Hospitals NHS Foundation Trust, Newcastle upon Tyne, UK; University of Cambridge School of Clinical Medicine, Cambridge, UK

**Keywords:** Cardiac mIBG, sympathetic innervation, DLB, MCI-LB, Lewy body disease

## Abstract

The aim of this study was to determine whether there was a significant change in cardiac [123I]-metaiodobenzylguanidine uptake between baseline and follow-up in individuals with mild cognitive impairment with Lewy bodies (MCI-LB) who had normal baseline scans. Eight participants with a diagnosis of probable MCI-LB and a normal baseline scan consented to a follow-up scan between 2 and 4 years after baseline. All eight repeat scans remained normal; however, in three cases uptake decreased by more than 10%. The mean change in uptake between baseline and repeat was −5.2% (range: −23.8% to +7.0%). The interpolated mean annual change in uptake was −1.6%.

Molecular imaging using [123I]-metaiodobenzylguanidine cardiac scintigraphy (cardiac mIBG) can demonstrate the integrity of myocardial post-ganglionic sympathetic nerve terminals. Uptake of the mIBG tracer in the myocardium has been shown to be reduced in patients with Lewy body diseases, including dementia with Lewy bodies (DLB)^1,2^ and mild cognitive impairment with Lewy bodies (MCI-LB),^3^ and is a diagnostic biomarker in consensus criteria for both disease stages.

We previously reported that 22 of 37 participants with probable MCI-LB in the Newcastle SUPErB study had reduced cardiac mIBG uptake.^4^ The remaining 15 participants meeting criteria for probable MCI-LB had baseline cardiac mIBG uptake within two standard deviations of that of healthy controls. Nine of these participants had baseline dopaminergic brain imaging within normal limits; six had abnormal results.

In this study, we rescanned participants with probable MCI-LB from the Newcastle SUPErB study who had normal cardiac mIBG uptake at baseline, after an interval of at least 2 years. We aimed to determine whether there was a significant change in cardiac mIBG uptake at follow-up and to identify the proportion of scans that changed from normal at baseline to abnormal at follow-up.

## Method

### Participants

The methods used for recruitment and assessment of participants have been detailed previously.^4^ Participants with a diagnosis of probable MCI-LB who had normal baseline cardiac mIBG and who still met criteria for probable MCI-LB at follow-up were approached and asked to consent to a repeat scan. One participant was on a different serotonin–norepinephrine reuptake inhibitor at repeat scan compared with baseline; all other participants had no changes in medications known to interfere with mIBG uptake.^5^

### Ethics statement

The authors assert that all procedures contributing to this work comply with the ethical standards of the relevant national and institutional committees on human experimentation and with the Helsinki Declaration of 1975, as revised in 2008. All participants gave written informed consent to participate. The study received ethical approval from the National Research Ethics Service Committee North East Newcastle & North Tyneside 2 (ID 15/NE/0420).

The lead author, G.G., affirms that the manuscript is an honest, accurate, and transparent account of the study being reported; that no important aspects of the study have been omitted; and that any discrepancies from the study as planned have been explained.

### Diagnosis

Consensus diagnoses of probable MCI-LB, possible MCI-LB or MCI due to Alzheimer's disease (MCI-AD) were made blinded to cardiac mIBG results, as described in our previous publications.^4,6^ Briefly, probable MCI-LB was diagnosed in participants with either two or more core features of Lewy body disease or at least one core feature with abnormal dopaminergic imaging. Participants were grouped as possible MCI-LB if they presented with only one core feature or abnormal dopaminergic imaging. Participants were grouped as MCI-AD if they had no core features, normal dopaminergic imaging and a decline characteristic of Alzheimer's disease.

### Imaging

Cardiac mIBG scans were acquired using the same procedure and scanner as at baseline.^4,6^ Briefly, this consisted of a 111 MBq [123I]-mIBG injection and planar imaging at 3–4 h following injection using medium energy collimators. The heart to mediastinum ratio (HMR) at baseline and follow-up was calculated using the processing method developed in previous work.^7^ The baseline and follow-up images were processed at the same time, by the same operator (G.G.).

### Analysis

HMR values were compared between baseline and repeat using a paired *t*-test, and the mean difference was recorded, with *P*-value and 95% confidence interval. The proportion of participants with repeat HMRs below the normal threshold of 1.85 was also recorded. The mean annual change in uptake was calculated using linear interpolation.

## Results

Of the 15 participants with probable MCI-LB and normal scans, four could not be approached for a repeat scan as they had progressed to DLB (an endpoint within the main study criteria). One participant had died, one had withdrawn from the study and one participant was unwilling to attend owing to concerns about contracting COVID-19. The remaining eight participants were scanned between November 2020 and June 2021 (Table 1).

**Table 1:** Participant diagnostic features and imaging biomarker results

	Pt 1	Pt 2	Pt 3	Pt 4	Pt 5	Pt 6	Pt 7	Pt 8
Age at baseline, years	68	69	60	69	82	72	78	76
Sex, male/female	Male	Male	Male	Male	Male	Male	Male	Male
Diagnosis at baseline	Prob MCI-LB	Prob MCI-LB	Prob MCI-LB	MCI-AD	Prob MCI-LB	Possible MCI-LB	Prob MCI-LB	Prob MCI-LB
Diagnosis at most recent annual clinical follow-up	Non-degenerative MCI	Prob MCI-LB	Prob MCI-LB	Prob MCI-LB	Prob MCI-LB	Prob MCI-LB	Prob MCI-LB	Prob MCI-LB
Time between baseline and repeat mIBG, months	49	27	38	36	37	57	52	27
Baseline core features	Cognitive fluctuations; RBD	Visual hallucinations; RBD	Cognitive fluctuations; RBD	None	Parkinsonism	RBD	Cognitive fluctuations; RBD	Parkinsonism; RBD
Core features at follow-up	n/a	Cognitive fluctuations; RBD	Cognitive fluctuations; RBD	Visual hallucinations; parkinsonism	Cognitive fluctuations; parkinsonism	Cognitive fluctuations; RBD	Cognitive fluctuations; RBD	Parkinsonism; RBD
New features since baseline?	n/a	No change	No change	Two new features	One new feature	One new feature	No change	No change
Potentially interacting medication^13^								
Baseline	Amlodipine	Venlafaxine	Nil	Mirtazapine, amlodipine	Venlafaxine	Citalopram	Tramadol	Nil
Repeat	No change	Duloxetine	No change	No change	No change	No change	No change	No change
Imaging results:								
Baseline mIBG HMR	3.25	2.53	3.04	2.13	2.58	2.79	2.52	3.69
Follow-up mIBG HMR	2.82	2.05	3.25	2.28	2.64	2.82	1.92	3.57
Percentage change	−13%	−19%	+7%	+7%	+2%	+1%	−24%	−3%
Baseline FP-CIT rating	Normal	Abnormal	Normal	Normal	Abnormal	Normal	Normal	Abnormal
Follow-up FP-CIT rating	Normal	Normal	Normal	Abnormal	Abnormal	Normal	Normal	Not done

FP-CIT, [123I]N-ω-fluoropropyl-2β-carbomethoxy-3β-(4-iodophenyl)nortropane ([123I]Ioflupane); HMR, heart to mediastinum ratio; MCI-LB, mild cognitive impairment with Lewy bodies; mIBG, [123I]-metaiodobenzylguanidine; RBD, rapid eye movement sleep behaviour disorder; Pt, participant.

The mean change in HMR between baseline and repeat was −5.2% (range: −23.8% to +7.0%; Table 1). The interpolated mean change over a 12-month period was −1.6%. No significant change was found between baseline and repeat HMR (*P* = 0.22), and none of the participants had abnormal follow-up mIBG scans. Three of the eight participants had an HMR change greater than 10% (all lower at follow-up).

## Discussion

In our previous publication, we reported that 41% of probable MCI-LB participants had normal cardiac mIBG scans at baseline; that is, they had an HMR within two standard deviations of the mean of local normal controls.^3^ Eight of these participants consented to be rescanned between 2 and 4 years later, and all eight scans remained within normal limits. The participant with the greatest change in uptake (−24%) had had a pacemaker fitted between baseline and follow-up. However, this is not expected to reduce sympathetic innervation or attenuate tracer signal.

The annual mean change in uptake in our participants was −1.6%, a lower rate of change than that seen in patients with Parkinson's disease. Watanabe et al^8^ conducted baseline and repeat cardiac mIBG imaging in 44 patients with Parkinson's disease, with a mean interval of 9 months. The mean change in cardiac uptake was −2.9%. However, as expected for a Parkinson's disease cohort, most scans showed low or absent uptake at baseline. Lamotte et al^9^ repeated [18F]-dopamine positron emission tomography in 31 participants with Parkinson's disease with a median follow-up of 3.5 years. They reported a mean annual change in cardiac uptake of −3% in patients with Parkinson's disease with orthostatic hypotension and −4% in Parkinson's disease patients without. We found no previous studies assessing change in cardiac sympathetic imaging in patients with either MCI-LB or DLB, so this study provides pilot data to inform the direction of future repeatability studies.

To estimate the longitudinal variability, we assumed that any increases in cardiac uptake in our study were random and reflected the repeatability. Two participants had increases in HMR, both of 7%. One participant, whose HMR dropped by 13%, has recently been assessed as not having a neurodegenerative disease after all. Excluding the two participants with large decreases in uptake of 19 and 24%, the mean change was 6%. This suggests that, in practice, there could be more variation in follow-up cardiac mIBG scanning than the 4% found by Bateman et al^10^ with repeat scanning after 1–2 weeks. In our recent publication on serial dopaminergic imaging,^11^ we also demonstrated higher test–retest repeatability than previously reported. This may have been because in our study repeat scans were done years after baseline and were designed to mimic typical clinical scans rather than following an unrealistic tightly controlled research protocol.

For five participants, there was no evidence of any decrease in cardiac mIBG uptake. These individuals may have no involvement of the cardiac sympathetic nervous system, or perhaps mild denervation that cannot yet be detected. The sensitivity of cardiac mIBG is known to be higher at the dementia stage.^2^ Raffel et al postulated that the molecular structure of the tracer makes cardiac mIBG imaging insensitive to mild to moderate changes in innervation.^12,13^ This suggests that planar [123I]-mIBG imaging may not be suitable for detecting subtle early changes in cardiac sympathetic function.

The key limitation of this extension to our main study was that we had no definitive reference standard for Lewy body disease, as no participants have yet donated brain tissue. Three participants had core clinical features of MCI-LB but normal scans at both baseline and follow-up. Their continued follow-up is of particular interest. Further limitations include the small all-male sample and that participants who had progressed to dementia could not be included. One participant was on a different antidepressant medication at repeat imaging compared with baseline; however, these were both the same serotonin–noradrenaline reuptake inhibitor class.

In conclusion, a decline in cardiac mIBG uptake compared with normal baseline imaging is seen in some individuals with MCI-LB, but the rate of change appears to be small. In our study, all follow-up scans remained within normal limits.

## Data Availability

The data and materials that support the findings of this study are available from the corresponding author, G.G., upon reasonable request.
